# Purkinje cells located in the adult zebrafish valvula cerebelli exhibit variable functional responses

**DOI:** 10.1038/s41598-021-98035-3

**Published:** 2021-09-15

**Authors:** Weipang Chang, Andrea Pedroni, Reinhard W. Köster, Stefania Giacomello, Konstantinos Ampatzis

**Affiliations:** 1 Neuroscience Department, Karolinska Institutet, 171 77 Stockholm, Sweden; 2 Cellular and Molecular Neurobiology, Zoological Institute, Technische Universität Braunschweig, Brunswick, Germany; 3 Science for Life Laboratory, KTH Royal Institute of Technology, Stockholm, Sweden

**Keywords:** Cerebellum, Spinal cord

## Abstract

Purkinje cells are critically involved in processing the cerebellar functions by shaping and coordinating commands that they receive. Here, we demonstrate experimentally that in the adult zebrafish valvular part of the cerebellum, the Purkinje cells exhibited variable firing and functional responses and allowed the categorization into three firing classes. Compared with the Purkinje cells in the corpus cerebelli, the valvular Purkinje cells receive weak and occasional input from the inferior olive and are not active during locomotion. Together, our findings expand further the regional functional differences of the Purkinje cell population and expose their non-locomotor functionality.

## Introduction

Locomotor behaviors, defined as a movement of the body through space, such as swimming, flying, or walking, are essential motor acts that give animals and humans the ability to move efficiently through their environment. Although locomotion might seem effortless, it is a complicated motor behavior that involves the activation of a large set of neurons^1–3^. Locomotion is one of many motor actions that the brain thoroughly controls. As such, the cerebellum plays a critical role in locomotion and maintaining motor coordination by recognizing neural patterns that it receives from various brain areas^4–8^. Accordingly, the canonical view regarding the Purkinje cells, the principal cerebellar neurons, predicts that they are rhythmically active during locomotion^7,9–12^. Recent evidence demonstrates the presence of functional and molecular diversity within the Purkinje cell population^12–16^. In particular, four functionally and morphologically distinct populations of Purkinje cells have been recognized to exist in the adult zebrafish corpus cerebellum, which is considered functionally comparable to mammalian spinocerebellum that display particular temporal activity during locomotion^12^. However, the degree to which regional differences exist in Purkinje cell and regulate locomotion remains unclear.

Zebrafish, as all actinopterygian fishes, possess an unusual anterior protrusion of the cerebellum reaching underneath the optic tectum of the posterior midbrain, and the function of this so-called valvula has remained a long-standing open question. The valvula has been studied extensively in mormyrid electric fish^17^, which is enormously enlarged and serves in the species’ electrosensory activity^18^. Thus, it is proposed to play a crucial role in the fish’s sensory-motor control^18,19^. However, it remains an understudied and enigmatic structure in fish species without an apparent electro-sensation, such as the zebrafish.

Here, we extended our previous study on adult zebrafish Purkinje cells^12^ by undertaking a thorough physiological characterization of the firing and the spontaneous activity patterns of those located in the valvula cerebelli. We reveal the presence of distinct Purkinje cell firing classes that also respond differentially to climbing fiber stimulation. In contradiction to the current view that requires most or all of the Purkinje cells to be active during locomotion, we show that none of the valvular cerebelli Purkinje cells discharge during swimming. This result further corroborates the functional difference of the valvula from the corpus cerebelli.

## Results

**Figure 1:**
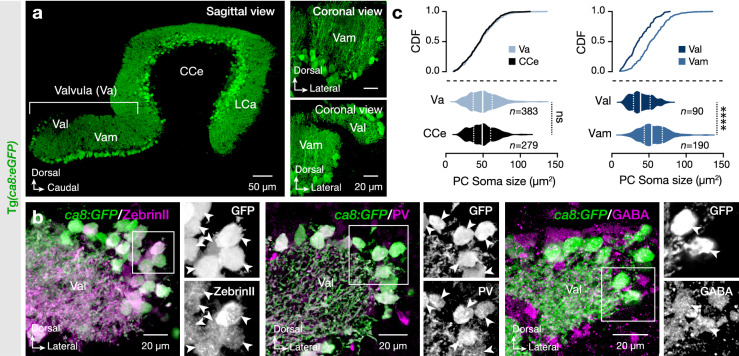
Expression pattern and specificity of *Ca8:eGFP* in the Purkinje cells of valvula cerebelli. (**a**) Sagittal and coronal sections show the specificity of the eGFP expression in all Purkinje cells (green) of the adult zebrafish cerebellum. (**b**) All eGFP-expressing Purkinje cells (green) are ZebrinII, PV, and GABA positive (magenta). Arrowheads indicate double-labeled cells. (**c**) Regionalized quantification of the Purkinje cell soma size in the adult zebrafish cerebellum. *CCe* corpus cerebelli, *CDF* cumulative distribution frequencies, *GABA* γ-aminobutyric acid, *LCa* lobus caudalis cerebelli, *GFP* green fluorescent protein, *PC* Purkinje cell, *PV* parvalbumin, *Va* valvular cerebelli, *Val* lateral part of valvular cerebelli, *Vam* medial part of valvular cerebelli. Data are presented as violin plots showing the median with quartiles. ****P < 0.0001; ns, not significant. For detailed statistics, see Supplementary Table S1.

To gain insight into the potential regional organization of the adult zebrafish cerebellum, we focused on the Purkinje cell population located in the less accessible region of the lateral part of the valvula cerebelli (Val), which is the anterior-most part of the cerebellar Purkinje cell layer (Fig. 1a)^20,21^. We ensured the selectivity of the *ca8:eGFP* line (*carbonic anhydrase 8*)^22^ to mark all the valvular Purkinje cells (Fig. 1b). We observed colocalization of the eGFP neurons with the Purkinje cell-selective markers ZebrinII, Parvalbumin, and GABA (Fig. 1b)^23^. Comprehensive systematic analysis of Purkinje cell soma size revealed a similar variability between the valvula (Va) and corpus (CCe) cerebelli (Fig. 1c). However, between the two sub-regions of the valvula cerebelli (Val, lateral; Vam, medial), we found a differential representation of Purkinje cells’ soma size (Fig. 1c).

**Figure 2:**
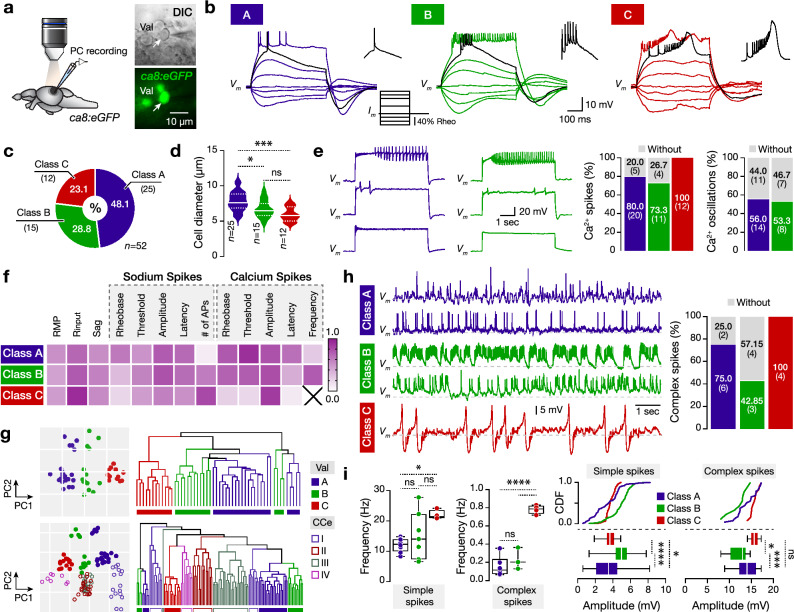
Variable firing, cellular, and spontaneous activity properties of the adult zebrafish valvular Purkinje cells. (**a**) Ex-vivo setup of an isolated intact brain from the Tg(*Ca8:eGFP*) line allows whole-cell patch-clamp recordings of valvular Purkinje cells. Arrow indicates a recorded cell. (**b**) The Purkinje cells show distinct firing patterns. Black trace shows the response at the rheobase. (**c**) Differential representation of the Purkinje cell firing classes in the adult zebrafish valvula cerebelli. (**d**) Valvula cerebelli Purkinje cells have different soma sizes. (**e**) Sample traces and quantification showing that part of firing class A and B Purkinje cells in valvula cerebelli fail to generate calcium-based spikes or repetitive calcium firing (oscillations). (**f**) Normalized mean values of the electrical properties detected for the valvular Purkinje cells are detailed in Supplementary Fig. S1*.* Normalizations were performed for each property to the highest obtained value. (**g**) PCA and hierarchical clustering plots depicting clusters of the valvular Purkinje cells (top) or valvular and corpus cerebelli Purkinje cells (bottom) based on physiological properties (as in (**f**)). Cells and data are colored by the assigned cell firing classification. (**h,i**) Sample traces showing the intense and variable spontaneous activity of the different Purkinje cell firing classes recorded in the adult zebrafish valvula cerebelli. Analysis of the presence, frequency and amplitude of simple and complex spikes between the Purkinje cells. *CCe* corpus cerebelli, *CDF* cumulative distribution frequencies, *DIC* differential interference contrast, *Rheo* rheobase, *RMP* resting membrane potential, *Rinput* input resistance, *Val* lateral part of valvular cerebelli. Data are presented as violin plots showing the median with quartiles and as box plots showing the median with 25/75 percentile (box and line) and minimum–maximum (whiskers). *P < 0.05; ***P < 0.001; ****P < 0.0001; *ns* not significant. For detailed statistics, see Supplementary Table S1.

Next, we examined the electrophysiological and firing properties of the Purkinje cells locating in the lateral part of the valvula cerebelli, using whole-cell patch-clamp electrophysiological recordings from the ex-vivo adult brain preparation (Fig. 2a)^12^. We detected that repetitive firing varied significantly between valvular Purkinje cells. In Fig. 2b we presented the valvular Purkinje cells firing patterns that we typically observed in response to depolarizing current steps injections. Using these variable firing responses of the valvular Purkinje cells we categorize them into three broad firing classes. Firing class A displayed irregular firing with high spike frequency adaptation; Firing class B discharged several action potentials with low or none adaptation; and firing class C displayed high firing rate with low calcium spike threshold resulted in a generation of mixed sodium and calcium spikes (Fig. 2b). Unbiased random recordings of Val Purkinje cells (*n* = 52) revealed the differential representation of the Purkinje cell classes in this cerebellar region (Fig. 2c). Also, we found a broad association between Purkinje cell firing type and soma size (Fig. 2d). Because a hallmark of all CCe Purkinje cells is to discharge numerous calcium-based spikes (oscillations) upon long and strong current step depolarizations^12,24^, therefore, we examined whether the valvular Purkinje cells possess this fundamental property. We found that while the majority (77.5%) of the A and B Purkinje cells could generate calcium-based spikes (Fig. 2e), with differences in their properties, yet 45% of them could not generate calcium action potential oscillations (Fig. 2e). Analysis of the Purkinje cell physiological properties revealed that the rheobase for the sodium spikes, the number of action potentials (sodium), the calcium spike threshold, and firing delay were significantly different between Purkinje cells in the valvula cerebelli (Fig. 2f, Supplementary Fig. S1).

Next, we confirmed the presence of functionally variable Purkinje cells in valvula cerebelli by Principal Component Analysis (PCA) and hierarchical clustering analysis using all the obtained physiological properties (as in Fig. 2f, Supplementary Fig. S1). The PC1 separated the valvular Purkinje cells into different clusters (Fig. 2g), similar to what we predicted using the firing pattern. Yet, the Purkinje cells that did not generate calcium-based spikes were segregated by PC2, suggesting a potential additional diversification of Purkinje cell into additional subpopulations (Fig. 2g). Probing possible associations between the Purkinje cells of the valvula and corpus cerebelli (CCe)^12^, we detected a partial but evident regional separation of Purkinje cell populations (Fig. 2g). Given the apparent similarities of the electrophysiological properties between all the adult zebrafish Purkinje cells (Val and CCe), we observed a cluster separation between them (Fig. 2g). Hierarchical clustering analysis revealed that the first split in the dendrogram gave one group of two clusters containing the A and B Purkinje cells (without calcium spikes) of the valvula cerebelli suggesting that they share less common electrophysiological properties with the rest of Purkinje cells (Fig. 2g). The clusters were also separated evidently when we visualized the data using PCA (Fig. 2g). Moreover, the dendrogram revealed additional major clusters, with the group C and IV to share similarities while they display distinctive repetitive firing (Fig. 2g). Our data collectively indicate that different sets of Purkinje cell exist in the adult zebrafish cerebellum with distinctive firing and physiological properties.

Similar to the Purkinje cells located in zebrafish corpus cerebelli^11,12,24,25^, the valvular Purkinje cells displayed high spontaneous activity by firing numerous action potentials in an irregular pattern (Fig. 2h, Supplementary Fig. S2a). In most of the recorder neurons, we observed two distinct types of events, the simple and complex spikes (Supplementary Fig. S2a) that differed significantly in both frequency and amplitude (Supplementary Fig. S2b). However, our analysis revealed that 31.6% of the valvular Purkinje cells did not generate the characteristic large-amplitude and low-frequency complex spikes (Supplementary Fig. S2c). To determine whether there are any differences in the spontaneous activity between the different classes, we observed that most of the class A (75%), a few of the class B (42.85%), and all the class C (100%) valvular Purkinje cells generated complex spikes (Fig. 2h). Moreover, detailed analysis revealed profound differences in simple and complex spike frequency and amplitude between the three firing categories of the valvular Purkinje cells (Fig. 2i).

**Figure 3:**
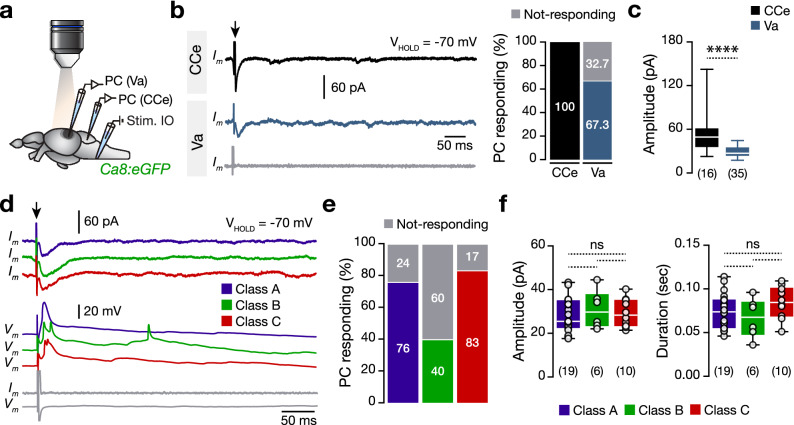
Direct electrical stimulation of inferior olive generates large-amplitude events in a proportion of valvular Purkinje cells. (**a**) Ex-vivo setup of an isolated intact brain from the Tg(*Ca8:eGFP*) line allows simultaneous whole-cell patch-clamp recordings of Purkinje cells and electrical stimulation of the inferior olive nucleus. (**b**) Voltage-clamp recordings of Purkinje cells showing the differential responses of the Purkinje cells located in corpus and valvula cerebelli. (**c**) Corpus cerebelli Purkinje cells responds to the inferior olive electrical stimulation, generating larger amplitude responses than the ones detected in the valvula cerebelli Purkinje cells. (**d**) Sample voltage- and current-clamp recordings show the responses of the adult valvular Purkinje cells to inferior olive stimulation pulse (Black arrow). Gray traces from a Purkinje cell that do not respond to inferior olive stimulation. (**e**) Quantification of the proportion of the valvular Purkinje cells that respond to the inferior olive stimulation. (**f**) Analysis of the recorded amplitude and duration between the Purkinje cells that respond to the inferior olive stimulation. *IO* inferior olive, *PC* Purkinje cell. Data are presented as box plots showing the median with 25/75 percentile (box and line) and minimum–maximum (whiskers). *ns* not significant. For detailed statistics, see Supplementary Table S1.

Previous characterization of the zebrafish Purkinje cell spontaneous activity suggested that the simple spikes were sodium-dependent action potentials likely arise from the parallel fibers, and the complex spikes were calcium-mediated spikes likely from climbing fibers^11,24,25^. Motivated by our findings, we explored the possibility that each valvular Purkinje cell firing class could receive differential input from the climbing fibers by electrically stimulating the inferior olive nucleus while recording from the ipsilateral Purkinje cells (Fig. 3a). We found that stimulation of the inferior olive nucleus produced large amplitude and broad responses to 67.3% of the valvular Purkinje cells (Fig. 3b). Compared to the CCe Purkinje cells that all reacted, the responses were significantly weaker in the Purkinje cells of the valvula cerebelli (Fig. 3c). Under voltage- and current-clamp recordings, our analysis revealed a similar proportion of the valvular Purkinje cell firing classes that responded to climbing fibers stimulation, generating similar amplitude and duration responses (Fig. 3d–f), with the ones that generate complex spikes during spontaneous activity (Fig. 2h), further suggesting that complex spikes in adult Purkinje cells resulted from the climbing fiber input as shown before in larvae zebrafish^11,25^.

**Figure 4:**
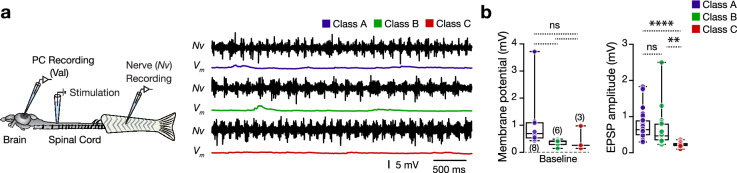
Adult zebrafish valvular Purkinje cell do not discharge during fictive locomotion. (**a**) Ex-vivo setup of the brain-spinal cord allows simultaneous recordings of Purkinje cells and ipsilateral motor nerves. Sample recordings from each valvular Purkinje cell firing class during locomotion. (**b**) Analysis of membrane potential depolarization and the amplitude of the recorded EPSPs during locomotion between the different classes of Purkinje cells. Data are presented as box plots showing the median with 25/75 percentile (box and line) and minimum–maximum (whiskers). **P < 0.01; ****P < 0.0001; *ns* not significant. For detailed statistics, see Supplementary Table S1.

Finally, to gain insight into the valvular Purkinje cells’ motor functionality, we examined whether the physiologically different classes of valvular Purkinje cells respond to spinal locomotor network activity. We performed whole-cell patch-clamp recordings on individual valvular Purkinje cells while recording motor nerve activity of the ipsilateral central pattern generator (CPG) in an ex-vivo preparation (Fig. 4a)^12^. Electrical stimulations (10 pulses, 1 Hz) of the brains descending axons induced a long fictive locomotor burst activity episode (Fig. 4a). Surprisingly, we observed that none of the valvular Purkinje cells were discharging during the ongoing swim episode (Fig. 4a). Also, we observed non-significant membrane depolarization changes between the valvular Purkinje cell firing classes. However, analysis of the recorded EPSPs showed that Purkinje cells belonging to class A and B received more substantial inputs than class C Purkinje cells during locomotion (Fig. 4b). Together our data indicate the role of the valvular cerebelli Purkinje cells to encode non-locomotor related functions.

## Discussion

Here, we expanded the investigations about adult zebrafish Purkinje cell functional diversity^12^ and demonstrated a striking physiological difference between corpus cerebelli and valvular cerebelli Purkinje cells, each forming their separate functional entities. Furthermore, we found that valvular Purkinje cells can be distinguished into specific firing activity classes indicating their distinct firing, cellular and functional properties. We also revealed that in clear difference to Purkinje cells of the corpus cerebelli, none of the valvular Purkinje cells responded to locomotion. While these Purkinje cells could still be dedicated to process solely cranial motor actions, we further revealed that a significant proportion of valvular Purkinje cells were void of receiving climbing fiber input from the inferior olive, strongly suggesting that these Purkinje cells do not serve locomotor functions and possibly are involved in processing non-locomotor related information. Hence, our data will render the opportunity to address locomotor and non-locomotor functions of cerebellar Purkinje cells in specific dedicated neuronal subtypes. Therefore, identifying genes that will be expressed differentially to discriminate between such locomotor and non-locomotor Purkinje cells will be critical.

The Purkinje cell population of the corpus cerebelli is well characterized in zebrafish^11,12,23–26^, and recently intra-population differences were recognized^11,12^. In comparison with the Purkinje cells located in the adult zebrafish corpus cerebelli, the valvular firing class C was detected for the first time. However, the valvular class C Purkinje cells shared several similarities in their electrophysiological properties with the corpus type IV Purkinje cells (bursting), suggesting two closely related groups of Purkinje cells with distinctive firing patterns and functions. Moreover, in the valvula, we did not observe the strong adapting Purkinje cell firing types found exclusively in the corpus cerebelli^12^. It is worth emphasizing that all the adult zebrafish Purkinje cells, besides the apparent distinct repetitive firing patterns we used in our classification here and before^12^, yet share numerous common electrophysiological properties. This is largely expected as all the Purkinje cells share a common developmental program, expressing the same transcription factors and molecular markers that have been analyzed so far^22,23,27^. Hence, it is essential to consider whether our results regarding the existence of different Purkinje cell categories will be supported by other evidence regarding the presence of selective molecular markers or differences in expression strength or timing that will further confirm and probably molecularly explain the functional differences presented here. Though the Purkinje cell layer continues seamlessly from the corpus to the valvula, these structures form cerebellar functional subcompartments built from different combinations of common and specific Purkinje cell subtypes. Recent evidence from the zebrafish genetic model of Spinocerebellar Ataxia Type 1 (SCA1) revealed an apparent preferential regional vulnerability of Purkinje cells that affected significant the exploratory behavior but not the animal’s locomotion^28^, supporting further the presence of regional specific Purkinje cell contributions to different cerebellar functions. Hence, our findings further suggest that multiple functionally diverse classes of Purkinje cells are essential for dividing the labor of the demanding computational duties of the cerebellum to process numerous and diverse locomotor and non-locomotor functions.

This observation could also account for inferior olivary neurons, because we found valvular Purkinje cells to receive weak input from the inferior olive in generating complex spikes, while complex spike generation in Purkinje cells of the corpus is much stronger and more reliable. Thus, either inferior olivary neurons innervating these different cerebellar subcompartments are heterogeneous or inferior olivary neurons synapse only sparsely or with reduced synaptic strength with valvular Purkinje cells. In the mormyrid fish valvula cerebelli, the climbing fibers fail to reach the molecular layer, suggesting a weak and sparse innervation that is received from the inferior olive^29^. However, in the zebrafish corpus cerebelli, the climbing fibers innervate the soma and the proximal dendrites of the Purkinje cells without intruding deeply into the molecular layer^23^; still, all the corpus cerebelli Purkinje cells responded to our inferior olive stimulation (Fig. 3b). A modular organization between particular inferior olive regions with the Purkinje cell zones, as defined by the expression of ZebrinII, exists in the mammalian cerebellum^14,30^. However, in zebrafish, all Purkinje cells are expressing ZebrinII^23^. Therefore, our data collectively suggest that if olivocerebellar modules are present in zebrafish, they could be defined either by the firing class or by the location of the Purkinje cells in the adult zebrafish cerebellum.

Besides the cerebellum's importance in locomotion^4–12,14^, accumulating studies revealed the cerebellum's key role in processing non-locomotor and non-motor related functions such as eye movement, predictions, perception, cognition, emotion, reward, and social behavior^31–35^. While in the mammalian cerebellum, such information is processed by Purkinje cells embedded in the regular cytoarchitecture, the valvula cerebelli in teleost fish could represent a separate specialized cerebellar module for processing such non-locomotor functions. This is evident from our recordings during a fictive locomotor episode, during which none of the valvular Purkinje cells were found to discharge. This is in contrast to Purkinje cells of the corpus cerebelli of which 72.4% fire during locomotion with temporal precision to swim cycle phases^12^. It is worth mentioning that type I Purkinje cells in the corpus cerebelli did not discharge during locomotion, similar to all valvular cerebelli Purkinje cells (A, B and C), suggesting that these neurons could share common functionalities. Future studies should be targeted to non-locomotor related Purkinje cells to reveal their precise function. In the past years, addressing social and motivational behavior in zebrafish has come into reach^36–38^. Thus, future studies that will selectively probe the valvular Purkinje cells’ activity will provide further insight into the regional- and class-specific role in the non-locomotor information processing of the cerebellar circuitry. Given the potential involvement of compromised cerebellar activity in autism spectrum disorder and attention deficit hyperactivity disorder^39–41^, such studies would be highly informative beyond our current enrichment of knowledge regarding the adult vertebrate cerebellum's architectural and functional complexity.

## Material and methods

### Experimental animals

dult transgenic *Tg(cpce-E1b:GFP)*^*bz13*^ known as *ca8:eGFP* zebrafish (*Danio rerio*) of both sexes (*n* = 147 animals; 8–10 weeks) were used. No selection criteria and blinding procedures were used to allocate zebrafish to any experimental group. The local Animal Research Ethical Committee (Karolinska Institutet, Stockholm, Sweden) approved all experimental protocols, Jordbruksverket (Ethical permit no. 9248-2017 and 19535-2020), and were implemented following EU guidelines for the care and use of laboratory animals (2010/63/EU) and guided according to the ARRIVE guidelines. All efforts were made to utilize only the minimum number of experimental animals necessary to obtain reliable scientific data.

### Immunohistochemistry

All animals were deeply anesthetized with MS-222 (Sigma-Aldrich, E10521). Then, the brains were carefully extracted and fixed in 4% paraformaldehyde (PFA) containing 5% saturated picric acid (Sigma-Aldrich, P6744) in phosphate-buffered saline (PBS) (0.01 M; pH 7.4, Santa Cruz Biotech., CAS30525-89-4) at 4 °C for 9-14 h, as described before^12^. Then tissue was cryoprotected overnight in 30% (w/v) sucrose in PBS at 4 °C, embedded in Cryomount (Histolab, 45830) sectioning medium, rapidly frozen in dry-ice-cooled isopentane (2-methylbutane; Sigma-Aldrich, 277258) at approximately − 35 °C, and stored at − 80 °C until use. Sagittal and coronal plane cryosections (thickness: 20–25 μm) of the tissue were collected and processed for immunohistochemistry^12^. Nonspecific protein binding sites were blocked with 4% normal donkey serum (NDS; Sigma-Aldrich, D9663) with 1% bovine serum albumin (BSA; Sigma-Aldrich, A2153) and 1% Triton X-100 (Sigma-Aldrich, T8787) in PBS for 1 h at room temperature (RT). Primary antibodies: Parvalbumin (mouse anti-PV, Swant 235, 1:2000), ZebrinII (mouse anti-zebrinII, a kind gift from Richard Hawkes, ACHR, University of Calgary, 1:400), GABA (Sigma-Aldrich, A2052, 1:2000) and GFP (rabbit anti-GFP, Molecular Probes, A-11122, RRID:AB_221569, 1:500 or chicken anti-GFP, Abcam, AB13970, RRID:AB_300798, 1:600) were diluted in 1% of the blocking solution and applied for 1–3 days at 4 °C. After thorough rinses with PBS, the tissues were then incubated with the appropriate secondary antibodies: anti-mouse (donkey, IgG-568, ThermoFisher A-10037, RRID:AB_2534013), anti-rabbit (donkey, IgG-488, ThermoFisher, A-21206, RRID:AB_2535792), anti-chicken (donkey, IgY-FITC, ThermoFisher, SA1-72000, RRID:AB_923386) diluted 1:500 in 1% Triton X-100 (Sigma-Aldrich, T8787) in PBS overnight at 4 °C. Finally, the tissue was thoroughly rinsed in PBS and cover-slipped with mounting solution (80% glycerol in PBS).

### Electrophysiological recordings

Recordings were performed in adapted ex-vivo preparation^12^ by unfolding part of the optic tectum to expose the valvula cerebelli. Specifically, adult zebrafish were cold-anesthetized in a slush of a frozen extracellular solution. The scalp and surrounding head tissue were removed carefully, and the preparation was transferred to a recording chamber that was continuously perfused with an extracellular solution containing 135.2 mM NaCl, 2.9 mM KCl, 1.2 mM MgCl_2_, 2.1 mM CaCl_2_, 10 mM HEPES, and 10 mM glucose at pH 7.8 (adjusted with NaOH) and an osmolarity of 290 mOsm. For whole-cell intracellular recordings of Purkinje cells in voltage- and current-clamp mode, electrodes (resistance ~ 15 MΩ) were pulled from borosilicate glass (outer diameter, 1.5 mm; inner diameter, 0.87 mm; Hilgenberg) on a micropipette puller (model P-97, Sutter Instruments) and filled with an intracellular solution containing 120 mM K-gluconate, 5 mM KCl, 10 mM HEPES, 4 mM Mg_2_ATP, 0.3 mM Na_4_GTP, and 10 mM Na-phosphocreatine at pH 7.4 (adjusted with KOH) and osmolarity of 275 mOsm. GFP positive Purkinje cells visualized with a fluorescent microscope (LNscope; Luigs & Neumann) equipped with a CCD camera (Lumenera) and were then explicitly targeted. Intracellular patch-clamp electrodes were advanced to the Purkinje cells using a motorized micromanipulator (Luigs & Neumann) while applying constant positive pressure. Intracellular signals were amplified with a MultiClamp 700B intracellular amplifier (Molecular Devices). All Purkinje cells were clamped at − 70 mV throughout the voltage-clamp recordings. All experiments were performed at room temperature (23–25 °C). Recorded spontaneous activity and measurement of the resting membrane potential (RMP) was in absence of any bias current. All other electrical properties quantified after the application of a bias hyperpolarization current to eliminate the spontaneous activity of the Purkinje cells. The current pulse injections (Fig. 2b) were performed as a fraction of the rheobase (minimum current to produce firing). For stimulation of the inferior olive, the electrode was pulled from borosilicate glass (1 mm outer diameter, 0.87 mm inner diameter) and polished. The stimulation electrode was placed in the ventral area of the caudal brain stem to cover the whole inferior olive nucleus (single strong pulse with duration of 1 ms).

### Fictive locomotion

For the evaluation of the activity of the valvular Purkinje cells during the activation of the spinal central pattern generators (CPGs; fictive locomotion), we used the adult zebrafish ex-vivo preparation developed previously^12^. Zebrafish were cold anesthetized in a slush of a frozen extracellular solution, then the scalp and adjacent tissue were gently removed to uncover the brain. Unfolding part of the optic tectum was necessary to expose the valvula cerebelli. The skin covering the body was gently removed to reveal all axial musculature. Most axial muscles were removed leaving intact the tail region. Extracellular recordings were performed from the motor nerves running through the intermyotomal clefts at the tail, where the musculature was left untouched. Activation of the locomotion was induced by extracellular stimulation (using a train of 10 pulses: 1 Hz) applied via a glass pipette placed at the junction between the brain and the spinal cord. All recordings were made from ipsilateral located Purkinje cells and motor nerves.

### Analysis

All immunofluorescence images of the adult zebrafish cerebellum were acquired using an LSM 800 laser scanning confocal microscope (Zeiss), with ZEN software (Zeiss) using a 20× or 40× (oil immersion) objectives. Purkinje cell soma sizes were measured using Fiji software^42^. All Purkinje cell electrical and cellular properties and events were detected and analyzed in a supervised fashion using Clampfit (version 10.6; Molecular Devices). All normalizations were performed for each individual property to the highest value for that particular feature. The number of sodium-based action potentials were quantified at 120% of rheobase current pulse injections. Cell diameter of the recorded Purkinje cells was measured directly in the electrophysiology setup in the unfixed preparations from the dorsal view of the valvula cerebelli. All figures, and graphs were prepared with Adobe Photoshop and Adobe Illustrator (Adobe Systems Inc., San Jose, CA, USA). Digital modifications of the images (brightness and contrast) were minimal to diminish the potential distortion of biological information. All double-labeled immunofluorescence images were converted to magenta-green to improve visualization of the results for color-blind readers.

### Dimensionality reduction and clustering analysis

We reduced the dimensionality of the electrophysiological parameters of the recorded cells (*n* = 52 Purkinje cells), by using the Principal Component Analysis (PCA; Prism, GraphPad Software Inc.). Hierarchical clustering across all cell classes was performed using the 13 cellular properties (Fig. 2f), without a priori assumptions that specific groups of neurons existed in our sample^12^. Using R, we first normalized and scaled the data. Subsequently, we calculated the distance matrix with the method “euclidean” and performed hierarchical clustering (hclust) with the “average” algorithm^12^.

### Statistical analyses

The statistical significance of differences between the means was gained using parametric tests (two-tailed unpaired or paired Student’s *t*-test, *one-way* ANOVA followed by post-hoc Tukey’s test) in Prism (GraphPad Software Inc.). The significance levels are indicated in the Figures as *P < 0.05, **P < 0.01, ***P < 0.001, ****P < 0.0001. Data are presented as mean ± standard error or as box- and violin-plots (median, 25th and 75th percentile, and minimal-maximal values as whiskers). Finally, the number (*n*) of used animals per group, Purkinje cells, or events are indicated in each figure panel.

## Supplementary Information

Supplementary Information.

## Data Availability

The descriptive statistics and detailed statistical analysis of the data supporting the conclusions are included in the supplementary information (see Supplementary Table S1). Raw data are available from the corresponding author upon request.
