# Supplementary material for: Purkinje cells located in the adult zebrafish valvula cerebelli exhibit variable functional responses
Source: Sci Rep. 2021 Sep 15;11:18408. doi: 10.1038/s41598-021-98035-3 (PMC8443705; doi:10.1038/s41598-021-98035-3)
Supplement: Supplementary file 1 — Supplementary Information. [file 41598_2021_98035_MOESM1_ESM.pdf]

Purkinje cells located in the adult zebrafish valvula cerebelli exhibit variable functional responses

Weipang Chang, Andrea Pedroni, Reinhard W. Köster, Stefania Giacomello and Konstantinos Ampatzis

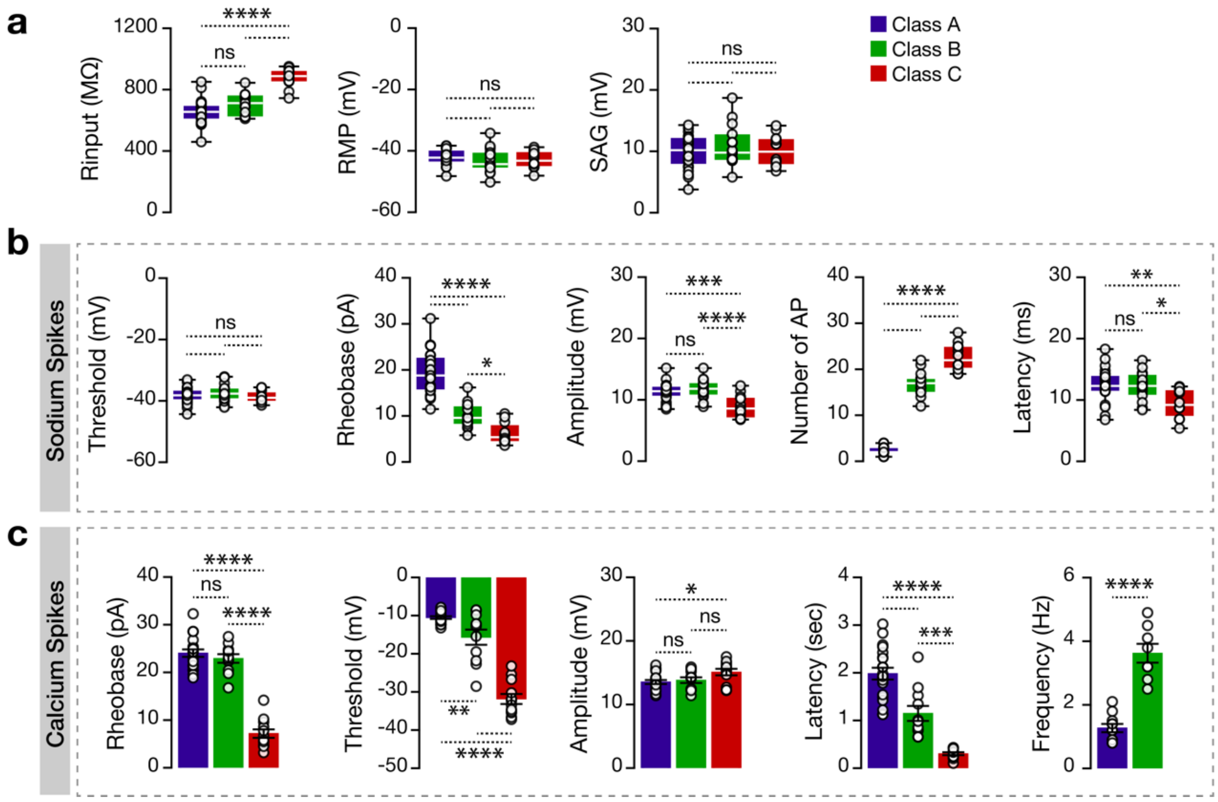

Supplementary Figure S1. Detailed analysis of the cellular and electrical properties of the valvular Purkinje cell firing classes. (**a**) General properties. (**b**) Features of the sodium-based spikes. (**c**) Properties obtained from calcium-based spikes. AP, action potential; RMP, resting membrane potential; Rinput, input resistance. Data are presented as means ± s.e.m. and as box plots showing the median with 25/75 percentile (box and line) and minimum–maximum (whiskers). **P*<0.05; ***P*<0.01; ****P*<0.001; *****P*<0.0001; ns, not significant. For detailed statistics, see Supplementary Table S1.

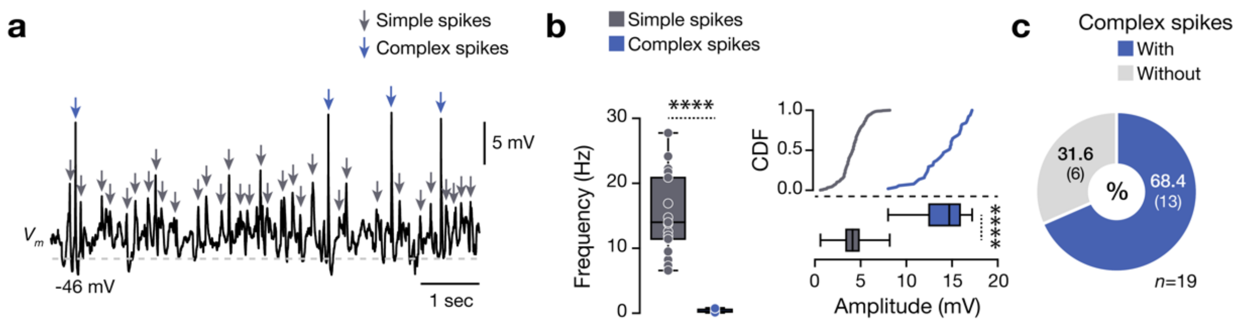

Supplementary Figure S2. Simple and complex spike events during the spontaneous activity of the valvular Purkinje cells. (**a**) Sample current-clamp recording of the spontaneous activity of a valvular Purkinje cell showing the detection of simple and complex spikes. (**b**) Analysis showing the differences in frequency and amplitude between the simple and complex spikes. (**b**) Not all the valvular Purkinje cells are able to generate complex spikes during the recorded spontaneous activity. CDF, cumulative distribution frequencies. Data are presented as box plots showing the median with 25/75 percentile (box and line) and minimum–maximum (whiskers). ****P<0.0001. For detailed statistics, see Supplementary Table S1.

Supplementary Table S1. Detailed statistics

Figure	Statistics	Result	Post-hoc Test	comparison	Significance	*P-value*
**1c<br>Left panel**	Unpaired *t*-test	t = 0.992, df = 660 (Two-tailed)		Va<br>Cce	ns	*P* = 0.3211
	Descriptive	Va (*n* = 279 Purkinje cells): 50.71 ± 1.072<br>Cce (*n* = 383 Purkinje cells): 49.37 ± 0.837				
**1c<br>Right panel**	Unpaired *t*-test	t = 7.504, df = 278 (Two-tailed)		Val<br>Vam	****	*P* < 0.0001
	Descriptive	Val (*n* = 190 Purkinje cells): 56.1 ± 1.268<br>Vam (*n* = 90 Purkinje cells): 40.34 ± 1.465				
**2d**	One-way ANOVA	$F_{(2, 49)}$ = 10.83, *P* = 0.0001	Tukey's test	A<br>B	*	P_{adj} = 0.018
				A<br>C	***	P_{adj} = 0.0001
				B<br>C	ns	P_{adj} = 0.2373
	Descriptive	A (*n* = 25): 7.856 ± 0.265<br>B (*n* = 15): 6.727 ± 0.311<br>C (*n* = 12): 5.95 ± 0.2806				
**2i<br>Simple spike frequency**	One-way ANOVA	$F_{(2, 16)}$ = 5.218, *P* = 0.018	Tukey's test	A<br>B	ns	P_{adj} = 0.4595
				A<br>C	*	P_{adj} = 0.0137
				B<br>C	ns	P_{adj} = 0.1113
	Descriptive	A (*n* = 8): 12.01 ± 0.803<br>B (*n* = 7): 15.19 ± 2.935<br>C (*n* = 4): 21.96 ± 0.758				
**2i<br>Complex spike frequency**	One-way ANOVA	$F_{(2, 10)}$ = 53.19, *P* < 0.0001	Tukey's test	A<br>B	ns	P_{adj} = 0.5141
				A<br>C	****	P_{adj} < 0.0001
				B<br>C	****	P_{adj} < 0.0001
	Descriptive	A (*n* = 6): 0.152 ± 0.044<br>B (*n* = 3): 0.231 ± 0.068<br>C (*n* = 4): 0.78 ± 0.023				
**2i<br>Simple spike amplitude**	One-way ANOVA	$F_{(2, 440)}$ = 76.68, *P* < 0.0001	Tukey's test	A<br>B	****	P_{adj} < 0.0001
				A<br>C	*	P_{adj} = 0.0421
				B<br>C	****	P_{adj} < 0.0001
	Descriptive	A (*n* = 103): 3.398 ± 0.151<br>B (*n* = 175): 4.863 ± 0.085<br>C (*n* = 165): 3.723 ± 0.04				
**2i<br>Complex spike amplitude**	One-way ANOVA	$F_{(2, 48)}$ = 8.416, *P* = 0.0007	Tukey's test	A<br>B	*	P_{adj} = 0.0377
				A<br>C	ns	P_{adj} = 0.0503
				B<br>C	***	P_{adj} = 0.0005
	Descriptive	A (*n* = 29): 14.01 ± 0.421<br>B (*n* = 9): 12.07 ± 0.739<br>C (*n* = 13): 15.63 ± 0.25				
**3c**	Unpaired *t*-test	t = 4.996, df = 49 (Two-tailed)	Va<br>CCe		****	*P* < 0.0001
	Descriptive	Va (*n* = 35): 29.33 ± 1.297<br>CCe (*n* = 16): 57.04 ± 7.788				
**3f<br>Amplitude**	One-way ANOVA	$F_{(2, 32)}$ = 0.167			ns	*P* = 0.8469
	Descriptive	A (*n* = 19): 28.79 ± 1.894<br>B (*n* = 6): 30.92 ± 3.568<br>C (*n* = 10): 29.39 ± 2.037				
**3f<br>Duration**	One-way ANOVA	$F_{(2, 32)}$ = 1.567			ns	*P* = 0.2243
	Descriptive	A (*n* = 19): 0.074 ± 0.004<br>B (*n* = 6): 0.066 ± 0.009				

		C (n = 10): 0.084 ± 0.005				
**4b**<br>**Membrane potential**	One-way ANOVA	$F_{(2, 48)}$ = 1.675			ns	P = 0.2227
	Descriptive	A (n = 8): 1.1 ± 0.385<br>B (n = 6): 0.365 ± 0.049<br>C (n = 3): 0.463 ± 0.26				
**4b**<br>**EPSP amplitude**	One-way ANOVA	$F_{(2, 86)}$ = 13.9,<br>P < 0.0001	Tukey's test	A<br>B	ns	P_{adj} = 0.4224
				A<br>C	****	P_{adj} < 0.0001
				B<br>C	**	P_{adj} = 0.002
	Descriptive	A (n = 48): 0.743 ± 0.053<br>B (n = 22): 0.624 ± 0.105<br>C (n = 19): 0.22 ± 0.014				
**Supplementary Figures**						
**S1a**<br>$\mathbf{R_{input}}$	One-way ANOVA	$F_{(2, 49)}$ = 38.66,<br>P < 0.0001	Tukey's test	A<br>B	ns	P_{adj} = 0.1143
				A<br>C	****	P_{adj} < 0.0001
				B<br>C	****	P_{adj} < 0.0001
	Descriptive	A (n = 25): 657.7 ± 15.64<br>B (n = 15): 705.8 ± 17.89<br>C (n = 12): 879.4± 17.95				
**S1a**<br>**RMP**	One-way ANOVA	$F_{(2, 49)}$ = 0.9193			ns	P = 0.4056
	Descriptive	A (n = 25): -41.98 ± 0.487<br>B (n = 15): -43.27 ± 1.035<br>C (n = 12): -42.86 ± 0.8				
**S1a**<br>**SAG**	One-way ANOVA	$F_{(2, 49)}$ = 0.5882			ns	P = 0.5592
	Descriptive	A (n = 25): 9.884 ± 0.542<br>B (n = 15): 10.89 ± 0.857<br>C (n = 12): 10.13 ± 0.721				
**S1b**<br>**Threshold**	One-way ANOVA	$F_{(2, 49)}$ = 1.017			ns	P = 0.3692
	Descriptive	A (n = 25): -38.17 ± 0.502<br>B (n = 15): -37.35 ± 0.768<br>C (n = 12): -38.71 ± 0.531				
**S1b**<br>**Rheobase**	One-way ANOVA	$F_{(2, 49)}$ = 60.18,<br>P < 0.0001	Tukey's test	A<br>B	****	P_{adj} < 0.0001
				A<br>C	****	P_{adj} < 0.0001
				B<br>C	*	P_{adj} = 0.0273
	Descriptive	A (n = 25): 19.31 ± 0.935<br>B (n = 15): 9.993 ± 0.691<br>C (n = 12): 6.133 ± 0.661				
**S1b**<br>**Amplitude**	One-way ANOVA	$F_{(2, 49)}$ = 11.71,<br>P < 0.0001	Tukey's test	A<br>B	ns	P_{adj} = 0.5337
				A<br>C	***	P_{adj} = 0.0005
				B<br>C	****	P_{adj} < 0.0001
	Descriptive	A (n = 25): 11.22 ± 0.299<br>B (n = 15): 11.78 ± 0.412<br>C (n = 12): 8.933 ± 0.528				
**S1b**<br>**AP number**	One-way ANOVA	$F_{(2, 49)}$ = 484.4,<br>P < 0.0001	Tukey's test	A<br>B	****	P_{adj} < 0.0001
				A<br>C	****	P_{adj} < 0.0001
				B<br>C	****	P_{adj} < 0.0001
	Descriptive	A (n = 25): 2.44 ± 0.153<br>B (n = 15): 16.93 ± 0.665<br>C (n = 12): 22.67 ± 0.846				
**S1b**<br>**Delay**	One-way ANOVA	$F_{(2, 49)}$ = 6.583,<br>P = 0.0029	Tukey's test	A<br>B	ns	P_{adj} = 0.5337

				A<br>C	**	P_{adj} = 0.0031
				B<br>C	*	P_{adj} = 0.0127
	Descriptive	A (n = 25): 12.48 ± 0.522<br>B (n = 15): 12.31 ± 0.595<br>C (n = 12): 9.483 ± 0.661				
**S1c<br>Rheobase**	One-way ANOVA	$F_{(2, 40)}$ = 107.8, P < 0.0001	Tukey's test	A<br>B	ns	P_{adj} = 0.6470
				A<br>C	****	P_{adj} < 0.0001
				B<br>C	****	P_{adj} < 0.0001
	Descriptive	A (n = 20): 24.05 ± 0.794<br>B (n = 11): 22.94 ± 0.918<br>C (n = 12): 7.192 ± 0.885				
**S1c<br>Threshold**	One-way ANOVA	$F_{(2, 40)}$ = 97.42, P < 0.0001	Tukey's test	A<br>B	**	P_{adj} = 0.0064
				A<br>C	****	P_{adj} < 0.0001
				B<br>C	****	P_{adj} < 0.0001
	Descriptive	A (n = 20): -10.5 ± 0.367<br>B (n = 11): -15.65 ± 1.983<br>C (n = 12): -31.83 ± 1.321				
**S1c<br>Amplitude**	One-way ANOVA	$F_{(2, 40)}$ = 3.986, P = 0.0264	Tukey's test	A<br>B	ns	P_{adj} = 0.8711
				A<br>C	*	P_{adj} = 0.0225
				B<br>C	ns	P_{adj} = 0.1323
	Descriptive	A (n = 20): 13.55 ± 0.308<br>B (n = 11): 13.84 ± 0.455<br>C (n = 12): 15.1 ± 0.516				
**S1c<br>Delay**	One-way ANOVA	$F_{(2, 40)}$ = 49.69, P < 0.0001	Tukey's test	A<br>B	****	P_{adj} < 0.0001
				A<br>C	****	P_{adj} < 0.0001
				B<br>C	***	P_{adj} = 0.0003
	Descriptive	A (n = 20): 1.983 ± 0.123<br>B (n = 11): 1.115 ± 0.157<br>C (n = 12): 0.305 ± 0.028				
**S1c<br>Frequency**	Unpaired *t*-test	t = 8.042, df = 17 (Two-tailed)		A<br>B	****	P < 0.0001
	Descriptive	A (n = 11): 1.273 ± 0.127<br>B (n = 8): 3.625 ± 0.297				
**S2b<br>Frequency**	Unpaired *t*-test	t = 8.768, df = 30 (Two-tailed)		Simple spikes<br>Complex spikes	****	P < 0.0001
	Descriptive	Simple spikes (n = 19): 15.28 ± 1.398<br>Complex spikes (n = 13): 0.363 ± 0.084				
**S2b<br>Amplitude**	Unpaired *t*-test	t = 48.78, df = 492 (Two-tailed)		Simple spikes<br>Complex spikes	****	P < 0.0001
	Descriptive	Simple spikes (n = 443): 4.098 ± 0.058<br>Complex spikes (n = 51): 14.08 ± 0.32				
